# Traumatic Macroglossia Associated With Clustered Epileptic Seizures in a Patient With Down Syndrome

**DOI:** 10.7759/cureus.116683

**Published:** 2026-09-21

**Authors:** Masataka Kitayama, Katsuhiko Kitazawa

**Affiliations:** 1 Department of Pediatrics, Asahi General Hospital, Asahi, JPN

**Keywords:** antibiotics, bite block, corticosteroids, down syndrome, seizures, tongue biting, traumatic macroglossia

## Abstract

Traumatic macroglossia is rare but can cause acute upper airway obstruction and become life-threatening, requiring prompt intervention. We report a case of traumatic macroglossia in a 30-year-old woman with Down syndrome and drug-resistant epilepsy, triggered by tongue biting during clusters of tonic seizures. The patient was admitted for seizure control; however, seizures persisted despite modification of antiseizure therapy. On hospital day 6, she developed rapidly progressive tongue swelling accompanied by fever. During observed seizures, the tongue was repeatedly caught between the dental arches. The tongue showed ulcerative lesions, purulent exudate, marked edema, and congestion. To prevent further injury, a custom-fabricated bite block was used. Empiric broad-spectrum antimicrobials, systemic corticosteroids, and antihistamines were initiated. With close collaboration among the oral surgery, otorhinolaryngology, and intensive care teams and with preparation for possible emergency airway intervention, conservative management was selected. Further optimization of antiseizure therapy reduced seizure frequency, followed by improvement in tongue swelling. The patient recovered without invasive airway management. This case illustrates that severe traumatic macroglossia can result from seizure-related tongue biting but may be managed without invasive airway intervention through careful airway assessment, mechanical protection with an effective bite block, and appropriate adjunctive pharmacotherapy.

## Introduction

Macroglossia is defined as a condition in which the tongue protrudes beyond the alveolar ridge or dental arch at rest [1]. Traumatic macroglossia refers to acute tongue swelling caused by trauma. It is potentially life-threatening because rapid enlargement of the tongue can result in upper airway obstruction and may be fatal without prompt airway management [2]. Traumatic macroglossia is most commonly associated with external trauma or surgical procedures, whereas cases caused by epileptic seizure-related tongue biting are rarely reported. Here, we report a case of traumatic macroglossia in a patient with Down syndrome and drug-resistant epilepsy, triggered by frequent tonic seizures accompanied by tongue biting, in whom invasive airway intervention was successfully avoided with conservative treatment.

## Case presentation

A 30-year-old woman with Down syndrome and drug-resistant epilepsy, who had been receiving ethosuximide, clonazepam, lacosamide, and lamotrigine, was admitted for treatment of frequent tonic seizures involving the face and both upper extremities. Each seizure lasted approximately 30 seconds and occurred more than 20 times per day. She was independent in activities of daily living, although complex conversation was difficult.

On admission, the patient was alert. Her vital signs were as follows: body temperature, 36.6°C; blood pressure, 112/70 mmHg; heart rate, 90 beats/minute; respiratory rate, 18 breaths/minute; and oxygen saturation, 99% on room air. No abnormal breath sounds were noted, and no tongue abnormality was observed. Laboratory findings on admission were unremarkable except for a mild elevation in C-reactive protein (Table 1). After admission, the dose of clonazepam was increased; however, adequate seizure control was not achieved. Therefore, continuous intravenous midazolam infusion was initiated on hospital day 3. From that night onward, oxygen desaturation accompanied by snoring during sleep was observed, and continuous positive airway pressure therapy was started. Because seizures remained uncontrolled despite continuous midazolam infusion, intravenous levetiracetam was added on hospital day 4. Meanwhile, given the concurrent use of multiple antiseizure medications, it was difficult to determine the efficacy of each agent during hospitalization. However, based on the patient’s clinical course during outpatient follow-up, lacosamide and lamotrigine were considered to have provided limited seizure control. Therefore, lacosamide was discontinued and lamotrigine was reduced. The dose of ethosuximide was also adjusted because of an elevated serum drug concentration. On hospital day 5, seizure frequency remained unchanged, but mild tongue swelling was noted. Midazolam was discontinued because of increasing somnolence; however, no change in seizure frequency was observed after its discontinuation. Tongue biting occurred during the seizures, although the patient’s respiratory status remained stable.

**Table 1:** Blood test results of the patient NA: not assessed.

Variables	On Admission	Hospital Day 6	Hospital Day 13	Reference Range
White blood cell count, /µL	4,700	10,100	4,300	3,300-8,600
Neutrophils, %	59.5	84.9	54.7	38.3-74.7
Hemoglobin, g/dL	13.0	13.1	12.9	11.6-14.8
Platelet count, ×10^3/µL	254	258	331	158-348
C-reactive protein, mg/dL	0.60	13.04	1.13	0.00-0.14
Alanine aminotransferase, U/L	18	14	22	13-30
Aspartate aminotransferase, U/L	17	12	46	7-23
Creatine kinase, U/L	26	132	28	41-153
Blood urea nitrogen, mg/dL	15	13	10	8-20
Creatinine, mg/dL	0.73	0.68	0.64	0.46-0.79
Sodium, mmol/L	140	136	138	138-145
Potassium, mmol/L	4.5	4.1	4.2	3.6-4.8
Chloride, mmol/L	106	101	103	101-108
Ammonia, µg/dL	60.8	NA	NA	18.7-86.9

On the morning of hospital day 6, marked tongue swelling was observed, followed later by fever up to 38.8°C. During seizures, the canine and upper premolar teeth were observed to bite into the tongue. Ulcerative changes with purulent discharge were present in the posterior third of the left side of the tongue, and the anterior two-thirds showed marked congestion (Figure 1). Laboratory testing showed elevated inflammatory markers, with a C-reactive protein level of 13.04 mg/dL and a white blood cell count of 10.1 × 10³/µL, with 84.9% neutrophils (Table 1). Physical examination, urinalysis, and chest radiography revealed no findings suggestive of a source of fever other than the tongue lesion. Contrast-enhanced computed tomography of the head, neck, and trunk showed no abnormal findings other than tongue enlargement (Figure 2). No abnormal density suggestive of intralingual hematoma or abscess formation was identified. 

**Figure 1:**
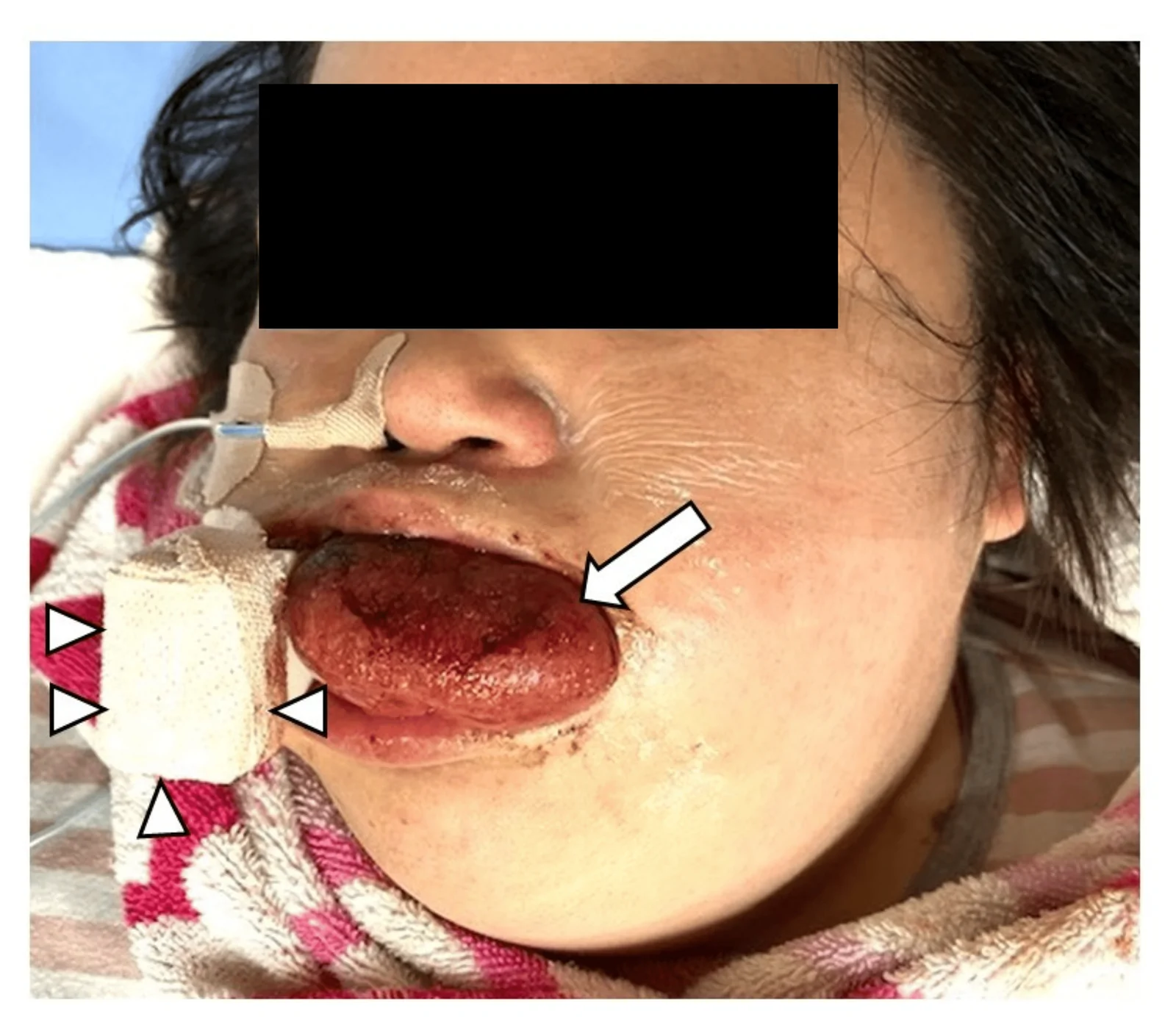
Appearance of the enlarged tongue The appearance of a markedly enlarged tongue (arrow) and a custom-made bite block (arrowhead) in a patient with Down syndrome.

**Figure 2:**
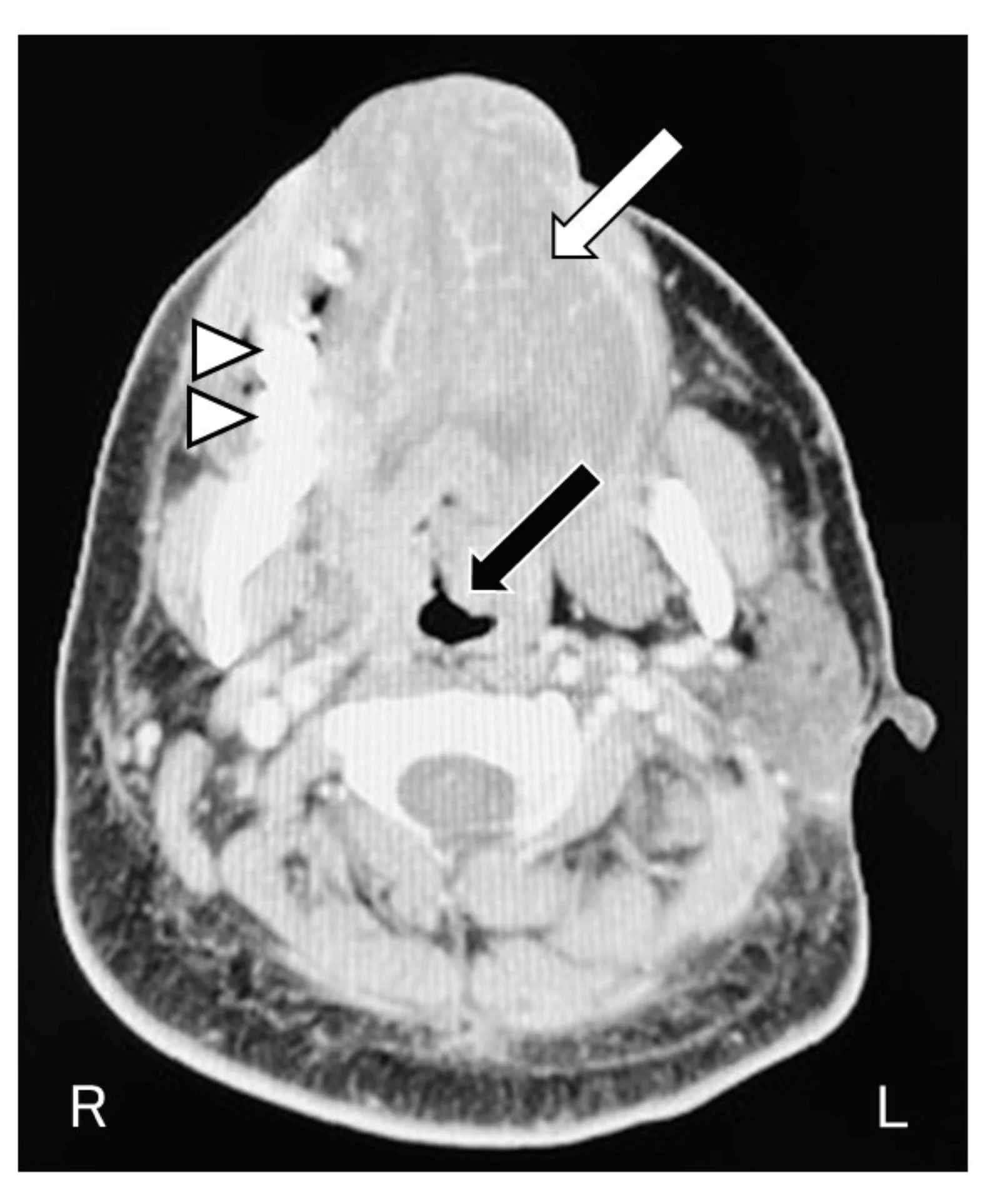
Contrast-enhanced axial computed tomography of the tongue Contrast-enhanced axial computed tomography demonstrates markedly enlarged tongue (white arrow) and a custom-made bite block (arrowhead). The upper airway is compressed but remains patent (black arrow).

Although no further decrease in oxygen saturation or inspiratory stridor was observed, auscultation revealed bilaterally diminished breath sounds. The differential diagnosis of macroglossia included traumatic swelling due to tongue biting, local infection, and angioedema. To prevent further tongue injury, a commercially available cylindrical bite block with a diameter of 10 mm was initially used. Intravenous ceftriaxone was started for suspected local tongue infection, prednisolone was administered for its anti-inflammatory effect, and chlorpheniramine maleate was given because angioedema remained a differential diagnosis. Because drug-induced angioedema was also considered, levetiracetam was discontinued. However, the commercially available bite block was insufficient to prevent tongue biting effectively during seizures. Therefore, an oral surgeon urgently fabricated a custom rectangular bite block using a rigid urethane dental bite block measuring 30 × 15 × 60 mm (height × width × length), which was wrapped with medical tape to provide cushioning. This device provided effective decompression and protection of the tongue. Frequent oral suctioning was required every few hours to prevent aspiration of saliva and airway obstruction.

The otorhinolaryngology and intensive care teams were consulted. Airway evaluation by laryngoscopy confirmed no swelling of the oral cavity, pharynx, or other soft tissues apart from the tongue. Although upper airway narrowing due to tongue swelling was present, the airway distal to the tongue was judged to remain patent. Accordingly, the patient was managed conservatively with spontaneous breathing under close observation, while preparations were made for endotracheal intubation and emergency tracheostomy if necessary.

The fever subsided on hospital day 7, and the macroglossia subsequently improved in parallel with a decrease in seizure frequency. No seizures were observed after hospital day 9. Purulent discharge from the tongue wound also resolved. Ceftriaxone and prednisolone were discontinued on hospital day 11, and the patient was discharged on hospital day 14. At discharge, the tongue had returned to approximately the same size as on admission. The overall clinical course is summarized in Figure 3.

**Figure 3:**
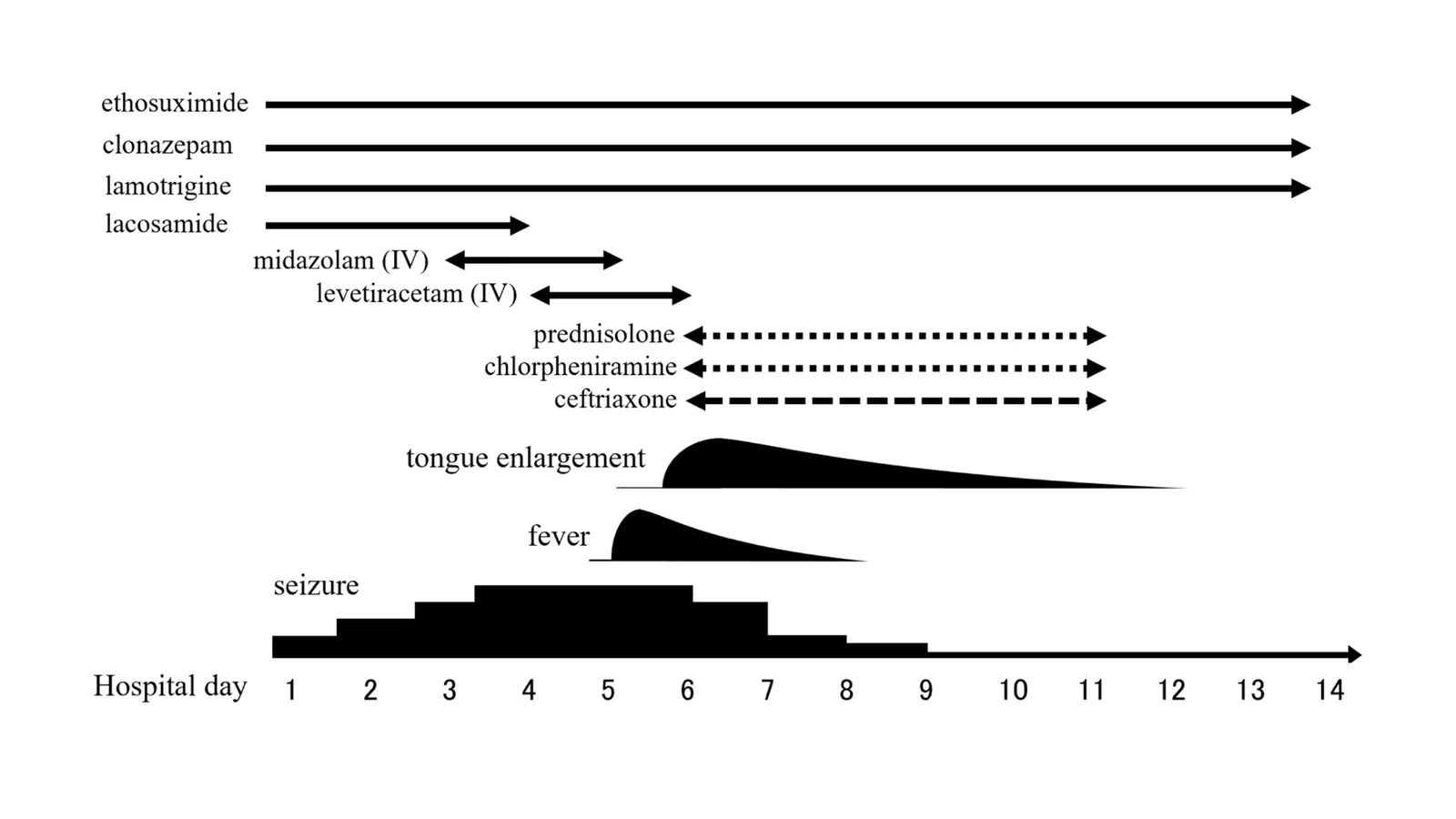
Clinical course after admission IV: Intravenous administration.

At follow-up in the oral surgery clinic two weeks later, the macroglossia had resolved, and the tongue ulcer was healing. No recurrence of macroglossia was observed during one year of follow-up.

## Discussion

In the present case, traumatic macroglossia developed in an adult patient with Down syndrome and drug-resistant epilepsy, precipitated by tongue biting during recurrent tonic seizures. Although tongue biting during epileptic seizures is well recognized [3], seizure-related traumatic macroglossia has rarely been reported [4,5]. In this case, conservative treatment was successful and allowed avoidance of invasive airway intervention, provided that careful airway assessment and preparation for emergency airway management were performed by a multidisciplinary team. The treatment strategy included antibiotics, corticosteroids, antihistamines, optimization of antiseizure therapy, and mechanical protection using a custom-fabricated bite block.

Traumatic macroglossia may progress rapidly and poses a substantial risk of airway obstruction; therefore, management should always prioritize airway protection. Previously reported causes include cleft palate surgery [6], tongue compression related to intraoperative positioning [7], and gunshot injury [8]. In the present case, local findings included laceration, ulceration, and purulent discharge, suggesting that superimposed local infection may have contributed to disease progression in addition to direct trauma from repeated tongue biting. These combined factors may have caused venous and lymphatic congestion, thereby exacerbating tongue swelling. In patients with Down syndrome, relative macroglossia is a frequent finding [9], which may have increased the patient’s susceptibility to tongue biting and subsequent traumatic macroglossia.

Drug-induced angioedema was also considered in the differential diagnosis because the patient had been receiving multiple antiseizure medications. Acute tongue swelling associated with clobazam has been reported [10], and levetiracetam, which had been newly introduced in this case, was also considered a possible culprit. Although levetiracetam-associated angioedema has been reported [11], this diagnosis was considered less likely in our patient because there was no lip or facial swelling, and the tongue showed clear traumatic and infectious features.

A notable feature of this case is that neither endotracheal intubation nor emergency tracheostomy was ultimately required. Avoidance of invasive airway intervention was made possible by close collaboration among the intensive care, otorhinolaryngology, and oral and maxillofacial surgery teams. This collaboration enabled repeated airway assessment, readiness for intubation, and prior identification of the field for emergency surgical airway access. Although evidence defining the optimal management of traumatic macroglossia caused by tongue biting is limited, interventions such as bite raisers to prevent further biting [12], careful repositioning of the tongue within the oral cavity [4], and intravenous corticosteroid therapy [13] have been reported to be useful. In the present case, the initial cylindrical bite block provided insufficient protection, whereas a larger and more stable rectangular bite block was effective in preventing further tongue injury. Intravenous antibiotics were also administered because signs of local infection were evident. Although it remains unclear which intervention contributed most to recovery, the combination of airway vigilance, mechanical protection, pharmacotherapy, and seizure control appeared clinically effective.

## Conclusions

Traumatic macroglossia should be recognized as a rare but potentially life-threatening complication of clustered epileptic seizures accompanied by tongue biting, particularly in patients with Down syndrome or pre-existing relative macroglossia. Early multidisciplinary evaluation is essential to assess airway patency, monitor for progression of upper airway obstruction, and prepare for urgent endotracheal intubation or surgical airway intervention if needed. In selected patients whose distal airway remains patent, conservative management with close airway monitoring, prevention of further tongue injury through the use of an effective bite block, optimization of seizure control, and adjunctive pharmacologic therapy may allow recovery without invasive airway intervention.
